# Nonmalarial Infant Deaths and DDT Use for Malaria Control

**DOI:** 10.3201/eid0908.030082

**Published:** 2003-08

**Authors:** Aimin Chen, Walter J. Rogan

**Affiliations:** * National Institute of Environmental Health Sciences, Research Triangle Park, North Carolina, USA

**Keywords:** DDT, DDE, malaria, breast-feeding, premature, infant, mortality, research

## Abstract

Although dichlorodiphenyl trichloroethane (DDT) is being banned worldwide, countries in sub-Saharan Africa have sought exemptions for malaria control. Few studies show illness in children from the use of DDT, and the possibility of risks to them from DDT use has been minimized. However, plausible if inconclusive studies associate DDT with more preterm births and shorter duration of lactation, which raise the possibility that DDT does indeed have such toxicity. Assuming that these associations are causal, we estimated the increase in infant deaths that might result from DDT spraying. The estimated increases are of the same order of magnitude as the decreases from effective malaria control. Unintended consequences of DDT use need to be part of the discussion of modern vector control policy.

After the Stockholm convention in 2001, which called for the gradual ban of the persistent pesticide dichlorodiphenyl trichloroethane (DDT), more than a dozen countries in sub-Saharan Africa requested exemptions for DDT use to control malaria (*1*). Discussions on the health consequences of DDT use have focused on reducing infant illness and death from vector control. The possibility of observable toxicity has been minimized because only a few studies show that DDT may affect child health and development (*2*–*5*). In laboratory experiments, effects of DDT include hepatic and central nervous system toxicity, estrogenic and antiandrogenic effects, and possible carcinogenicity (*6*,*7*). Some epidemiologic evidence suggests that DDT exposure increases preterm delivery and small-for-gestational-age births (*8*) and shortens the duration of lactation (*9*,*10*); these conditions could increase the rate of infant deaths (*11*,*12*) and thus attenuate any benefits on mortality rates from a reduction in malaria. While the observed associations between DDT and such outcomes might not be causal, the studies are not so flawed that the observations can be dismissed out of hand. We attempted to estimate the consequences for infant deaths if maternal DDT exposure in fact increases preterm births and decreases the duration of lactation with the strength of association seen in North America. If the associations are causal but the estimated effect on death rates is very small compared to the plausible benefits from vector control, then whether the associations are causal does not impact public health decisions. If, on the other hand, the estimated increases in infant death rates are similar to or larger than the expected benefits, whether the association is causal matters a great deal, and further investigation is warranted, especially in areas where DDT is reintroduced.

Although DDT can be found in the lipid of human tissues worldwide, and consequently in the fat of breast milk (*13*), levels of DDT and its metabolites in breast milk are much higher in areas where this insecticide has been applied for malaria control (*14*). Here, we use published data on the relationship between DDT spraying and levels in maternal serum and breast milk in Africa to estimate the increased exposure from spraying. We assume that, to obtain the benefit of reduced risk for malaria in the infant, the mother’s home must be treated and she must be exposed. We then estimate the effect of that exposure on the frequency of preterm births and on duration of lactation. We assume these relationships to be causal. Whether they are causal, and, if they are, whether the strengths of association seen in North America would occur in Africa is not known. Using infant-mortality rates specific to preterm births, or odds ratios for infant deaths by month-specific breast-feeding status, we estimated deaths attributable to the changed preterm birth rate and to the shortened duration of lactation that we assume would be caused by spraying DDT.

## Materials and Methods

The Medline database was searched for literature on DDT home spraying for malaria control and its effect on DDT concentration in serum samples or breast milk; DDT levels in blood or milk and pregnancy outcome or lactation duration; and pregnancy outcome or duration of lactation and infant deaths. Published data were reviewed and reanalyzed, if necessary, to estimate our hypothesized increase of infant deaths from DDT home spraying, consequent to small, early births and shorter lactation.

## Results

### Effect of Spraying on DDT in Serum and Breast Milk

Three studies on DDT levels in serum or breast milk from Kwa-zulu after DDT application for malaria control showed much higher DDT, DDE (dichlorodiphenyl dichloroethene, the most stable and persistent form of DDT), and DDD (dichlorodiphenyl dichloroethane) levels in the DDT-exposed group (*14*–*16*). Dwellings in the exposed area were treated with DDT for vector control (*14*). In the DDT-treated area, DDE concentration in serum samples was 103±85 μg/L; in the control area, the concentration was 6±7 μg/L (*16*). The median DDE levels in breast milk fat in the treated area (5.2–7.7 mg/kg) were all much higher than those in the control area (0.38–0.59 mg/kg) (*14*).

### DDE and Preterm Birth

A study based on the U.S. Collaborative Perinatal Project, which included 361 preterm births (<37 completed weeks’ gestation) out of 2,380 births (a rate of 151 per 1,000 births), indicated that the adjusted odds ratios (ORs) of preterm birth increased steadily with increasing concentration of maternal serum DDE (ORs=1, 1.5, 1.6, 2.5, 3.1 for DDE concentrations <15, 15–29, 30–44, 45–59, >60 μg/L, respectively) (*8*). The increase in serum DDE levels from spraying is greater than the range seen in the United States; the adjusted OR for the most highly exposed U.S. group is 3.1, but we used a more modest increase for this analysis (see below).

### Preterm Delivery and Infant Death

The preterm delivery rate in sub-Saharan Africa ranged from 5% to 22% (*17*–*20*) in studies from the 1990s, when DDT was not used or used only in small amounts. A Malawi study (*18*) showed that children born preterm had a crude relative risk (RR) of 2 for infant death; preterm birth accounted for 17% of infant deaths. Malaria itself might increase preterm birth, but this factor is counted in the contribution of malaria to infant deaths (see below). The observed RR of 2 in Malawi is lower than that seen in the United States and Canada, where mild (birth at 34–36 gestational weeks) and moderate (birth at 32–33 gestational weeks) preterm births were linked to a >2.9 fold increase in infant deaths (*12*). If we assumed that DDT use increased the overall preterm delivery rate from 15% (the midrange of the African rates) before spraying to 25% after spraying (RR 1.7, well below the 3.1 seen in U.S. data), and the RR of preterm birth for infant death is 2.0, we estimated a 9% (=((p_2_*RR+1-p_2_)-(p_1_*RR+1-p_1_))/(p_1_*RR+1-p_1_), p_1_=15%, p_2_=25%, RR=2) increase in total infant deaths.

### DDE and Duration of Lactation

**Figure 1:**
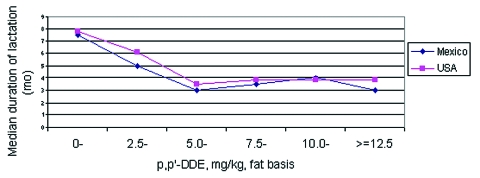
Levels of dichlorodiphenyl dichloroethene (DDE, the most stable and persistent form of DDT), in breast milk and duration of lactation.

Two birth cohort studies on DDE level in breast milk and duration of lactation both showed a negative relationship between DDE level and breast-feeding duration, whether in North Carolina (*10*) or Mexico (*9*). Figure 1 shows similar trends in the decrease of duration of lactation from both of these geographic sites. With a breast milk p,p’-DDE level of 5.0–7.5 mg/kg (fat basis), the median duration of breast-feeding is expected to be 3–4 months, down 40% to 50%, as compared with 7–8 months if p,p’-DDE level falls into the 0–2.5 mg/kg category.

Pooled analysis of data from 17 African countries from the World Fertility Surveys and Demographic and Health Surveys (DHS) in late 1970s and 1980s, when DDT was not used in most of Africa, showed that the mean duration of breast-feeding was 18.1 months (first quartile 12.0 months, median 18.8 months, and third quartile 23.7 months) (*21*). Another DHS dataset indicated the median breast-feeding duration in Africa from 1986 to 1990 was 19.3 months (*22*). Thus, if we assume the proportional decrease in duration of lactation attributable to high DDE concentration in milk fat in Africa is similar to that seen in North America, where we observed 40% shorter duration of lactation in women with approximately 6 mg/kg compared to women with approximately 0 mg/kg, and the result of spraying is to increase median DDE in milk fat from 0.4–0.6 mg/kg to 5–8 mg/kg (see above), the median expected duration of breast-feeding in areas with routine DDT application for malaria control should be approximately 11–12 months. The assumption of a similar relative decrease was the simplest. Alternatively, at one extreme, we could assume an absolute decrease of 5 months, from 19 to 13, as we progressed from low to high exposure (Figure 1); at the other extreme, since the median duration of lactation in Mexico was longer than that in North Carolina, but the DDE-level specific durations were the same, we could assume a median duration of 3 to 4 months at the high level of exposure in Africa as well.

### Shorter Lactation and Infant Deaths

**Figure 2:**
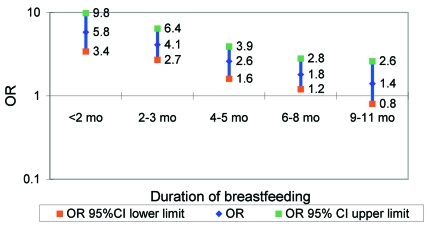
Protection of breast-feeding against infant death caused by infectious disease (not breast-feeding versus breast-feeding). CI, confidence intervals; OR, odds ratio. Source: World Health Organization study team.

Shorter duration of lactation increases the risk for infant and childhood deaths in both industrialized and developing countries (*23*–*25*). The World Health Organization (WHO) conducted a Mantel-Haenszel pooled analysis to review the effect of breast-feeding on infant and child death rates (*11*). The analysis identified breast-feeding as a strong protective factor against infant death, especially that caused by infectious illnesses such as diarrhea and acute lower respiratory tract infection. Breast-feeding was most protective in younger infants; nevertheless, it was still protective at 9 to 11 months after birth (Figure 2). If the median duration of lactation were shortened from 19 months to 11–12 months because of high concentrations of DDE, we would expect the proportion of children weaned before 12 months of life to increase from ~25% to 50%. In Africa, where prolonged breast-feeding is the norm, the risk of not being breast-fed continues into the second year of life, with ORs ranging from 8 in Ghana to 2 in Senegal (*11*). (Death after 1 year of age is no longer considered an infant death, but ORs should not change abruptly between 12 and 19 months of age). In the WHO analyses, the ORs for breast-feeding longer >1 year all were from Africa, and the ORs for breast-feeding <1 year were from Asia or South America. To estimate the effect of decreasing from a median duration of 19 months in Africa to 11 months, we used the most stable African estimate, from Senegal, which is 2.9 at 19 months, and compared it to the pooled estimate from Asia and South America at 11 months, which is 1.4. Thus, if we assume the overall RR of infant death from this degree of DDT-induced shortened lactation to be approximately 2.0, shortened lactation would result in a 20% (=((p_2_*RR+1-p_2_)-(p_1_*RR+1-p_1_))/(p_1_*RR+1-p_1_), p_1_=25%, p_2_=50%, RR=2) increase in infant mortality caused by infectious diseases.

### Increased Rate of Infant Death from Preterm Birth and Shorter Lactation

The reported infant mortality rate (IMR) of sub-Saharan African countries was 108/1,000 in 2000, 110/1,000 in 1995, and 111/1,000 in 1990 (*26*). If DDT use increases the IMR by 9% because of preterm delivery, IMR will increase by approximately 9.7/1,000 (=108*9%/1,000). Infectious diseases account for more than half of all infant deaths in Africa (*27*,*28*); thus, if DDT use increases IMR attributable to infectious disease by 20% (through shorter breast-feeding), another 10.8/1,000 (=(108/2)*20%/1,000) will be added to the IMR in areas with continuous DDT application for malaria control. The results would be a total estimated excess of 20.5/1,000 in IMR. On the other hand, maternal malaria caused 3% to 8% of all infant deaths in areas of Africa with stable malaria transmission (*29*). Malaria itself caused 20% of deaths in children <5 years of age (175/1,000 [*30*]) in Africa (*31*); malaria-specific infant deaths were estimated to be approximately 30% of such deaths (*32*).

## Discussion

When we combine data from North America on preterm delivery or duration of lactation and DDE with African data on DDT spraying and the effect of preterm birth or lactation duration on infant deaths, we estimate an increase in infant deaths that is of the same order of magnitude as that from eliminating infant malaria. Therefore, the side effects of DDT spraying might reduce or abolish its benefit from the control of malaria in infants, even if such spraying prevents all infant deaths from malaria. However, no studies from sub-Saharan Africa show that DDE shortens the duration of breast-feeding there specifically, and studies showing the relationship in the United States and Mexico both come from our group. Neither replications nor replication failures have been reported from other groups with longitudinal data. The relationship has a biologically plausible mechanism, in that both isomers of DDE are weak estrogens (*33*,*34*), and estrogen inhibits the stimulatory effect of prolactin on milk synthesis. (A woman’s own estrogen production is at its lowest postpubertal level at the beginning of lactation.) Older birth control pills with higher estrogen levels were linked to a decrease in milk volume or shortened duration of lactation (*35*).

In an attempt to replicate the finding on duration of lactation and DDE, Bouwman enrolled lactating women in a cross-sectional study comparing levels of DDE in milk in areas using and not using DDT for malaria control. Even though he did not follow children over time, this researcher reasoned that, if DDE shortened lactation, children of lactating mothers who had higher DDE levels would be, on the average, younger. He found that the DDE levels were much higher in the area where DDT was used than in a control area, but the age of children and the number of women unable to give a milk sample did not differ. This study is informative but cannot be interpreted as a failure to replicate the duration finding, since women were not followed over time, and, obviously, only women still lactating were eligible (*15*). Roberts argues against the causal nature of the association by observing that, in routinely collected data from Belize, rural women, who might be exposed to DDT, breast-fed longer than urban women, who probably were not exposed (*36*). However, these women are otherwise probably not similar (e.g., in their occupations or socioeconomic status). Nor is the classification of exposure exact enough to detect even very large effects of DDE. No studies in Africa show a relationship between DDE and preterm birth. The findings from the U.S. Collaborative Perinatal Project, while a confirmation of previous, smaller studies, still need to be replicated in Africa.

Another weakness of this analysis for current day sub-Saharan Africa is that the contrasts in mortality rates between breast-fed and bottle-fed children are derived from older data, when the impact of HIV on infant deaths was lower. In 1990, HIV caused 2% of deaths of children <5 years of age, whereas by 1999 it caused almost 8%. Five countries (Botswana, Namibia, Swaziland, Zambia, and Zimbabwe) had HIV-attributable under-5 death rates of approximately 30 per 1,000 (21% to 42% of total under-5 mortality). An additional 16 countries had HIV-specific under-5 mortality rates between 10 and 25 per 1,000 (8% to 20% of total under-5 death rates). The remaining 18 countries had rates <10 per 1,000 (0.1% to 6.5% of total under-5 deaths) (*37*). The effect of changing the duration of breast-feeding on HIV mortality is hard to quantify because, while any breast-feeding may increase transmission from infected mothers to infants, prolonged, exclusive breast-feeding may decrease deaths and HIV transmission (*38*). Such nonlinearities in dose-effect are difficult to reduce to the relatively simple formulae required for the kind of analysis presented here. Our conclusions are robust if shorter duration of lactation does not eliminate transmission of HIV and consequent deaths; even then, HIV mortality rates are likely only high enough in the five most affected countries to cancel the protective effect of breast-feeding entirely.

Our estimation provides a general framework of risk evaluation in sub-Saharan Africa. However, the variation in malaria transmission, illness, death, DDT spraying strategy, incidence of preterm birth, and duration of lactation should be kept in mind before the estimate is applied to a specific country or area. Since we focused on infant deaths, the benefits of DDT to child or adult malaria-specific deaths were not taken into account. Similarly, other potential adverse effects to humans and the environment that might occur from DDT spraying were not considered. However, this analysis counters the assumption that the risk of DDT use is unlikely to outweigh the benefit (*39*) and requires that such an assumption be tested.

The prohibition of DDT use for malaria control was probably not the sole cause of increasing malaria burden in sub-Saharan Africa (*40*), and thus DDT will probably not be the sole cure for the malaria epidemic there. Insecticide-treated bed nets, widely used in African households to prevent mosquito bites, are effective (*41*,*42*). Synthetic pyrethroid insecticides, cheaper than DDT, are available (*43*,*44*). Where DDT is used, all infant deaths, plus birth weights and the duration of lactation, should be counted. Some thought could also be given to a formal trial, since the risk and benefit calculations apply to individual dwellings, and an effective alternative, namely bed nets, is available.
